# Patient Race, Reported Problems in Using Glaucoma Medications, and Adherence

**DOI:** 10.5402/2012/902819

**Published:** 2012-10-15

**Authors:** Betsy Sleath, Susan J. Blalock, David Covert, Asheley Cockrell Skinner, Kelly W. Muir, Alan L. Robin

**Affiliations:** ^1^Division of Pharmaceutical Outcomes and Policy, Eshelman School of Pharmacy, University of North Carolina at Chapel Hill, Chapel Hill, NC, USA; ^2^Sheps Center for Health Services Research, University of North Carolina at Chapel Hill, CB No. 7590, Chapel Hill, NC 27599-7590, USA; ^3^Health Economics and Outcomes Research, Alcon Research Ltd., Fort Worth, TX, USA; ^4^Departments of Pediatrics and Health Policy and Management, University of North Carolina at Chapel Hill, Chapel Hill, NC, USA; ^5^Department of Ophthalmology, School of Medicine, Duke University and Durham VA Medical Center, Health Services Research and Development, Durham, NC, USA; ^6^Department of International Health, Bloomberg School of Public Health and Department of Ophthalmology, School of Medicine, Johns Hopkins University, Baltimore, MD, USA; ^7^University of Maryland School of Medicine, Baltimore, MD, USA

## Abstract

*Objective*. The objectives of the study were to (a) describe various factors potentially related to objectively measured adherence to glaucoma medications and self-reported glaucoma medication adherence self-efficacy and (b) examine the relationship between patient race, the number of patient reported-problems, and adherence in taking their glaucoma medication. This was a cross-sectional study conducted at two glaucoma subspecialist referral ophthalmology practices. *Methods*. We measured subjects' reported problems in using glaucoma medications, adherence to glaucoma medications utilizing the Medication Events Monitoring System (MEMS) devices, and general glaucoma medication adherence self-efficacy using a previously validated 10-item scale. Multivariable logistic and linear regression was used to analyze the data. *Results*. Seventy-one percent of patients self-reported at least one problem in using their glaucoma medications. White patients were more than 3 times more likely to be 80% adherent in using their glaucoma medications than non-White patients. Patients who had glaucoma longer reported significantly higher glaucoma medication adherence self-efficacy. Patients who reported more problems in using their medications had significantly lower glaucoma medication adherence self-efficacy. *Conclusions*. Eye care providers should assess patient reported problems and glaucoma medication adherence self-efficacy and work with patients to find ways to reduce the number of problems that patients experience to increase their self-efficacy in using glaucoma medications.

## 1. Introduction

Between 9% and 12% of all blindness in the Unites States is attributed to glaucoma [1]. Proper use of glaucoma medications can lower intraocular pressure and reduce the progression of glaucoma [2]. However, nonadherence to treatment regimens remains a significant problem [3, 4]. Approximately half of all subjects who are started on glaucoma medications will discontinue treatment within 6 months [5].

 Few prior studies have examined the relationship between race and adherence to glaucoma medications and these studies had contradictory results [3, 6–10]. Both Patel and Spaeth and Sleath et al. [7, 9] found that African Americans were more likely to report missing doses of their glaucoma drops than Whites, and electronic monitoring revealed African American race as a risk factor for poor glaucoma medication adherence [6]. However, other studies using electronic monitors [10] and pharmacy report [8] found no association between race and adherence to glaucoma medications. Racial differences in prescription adherence have been found in other therapeutic areas such as asthma, depression, and cardiovascular disease [11–13]. Nonadherence among African Americans may be an important contributing factor as glaucoma remains the leading cause of blindness in African Americans, whereas it is the second leading cause among Whites in the United States [14].

 Investigations using electronic medication monitors suggest that, on an individual basis, providers' estimates of patients' adherence correlate poorly with measured adherence [15]. However, patients' self-reported confidence in their ability to adhere to the prescribed medication regimen as measured by a self-efficacy survey correlates well with adherence metrics captured by the electronic monitor [10]. As such, survey tools such as the self-efficacy survey may help providers identify patients who are poorly adherent.

 In order to improve medication adherence, it would be helpful to identify specific problems related to glaucoma medication use for an individual patient. Accordingly, the purpose of this study is twofold. First, we describe the different types of problems patients on glaucoma medications report having when taking their medications. Second, we examine the relationship between both a patient's race and the number of patient-reported problems in taking their glaucoma medications and (a) whether patients are 80% or more adherent to their medications according to electronic monitors and (b) patient scores on the glaucoma medication adherence self-efficacy scale.

## 2. Methods

 This study was conducted at two referral-based glaucoma subspecialty practices in the mid-Atlantic region of the United States, one private practice and the second a clinic at an academic medical center. We used a cross-sectional design. Charts were prescreened by study staff prior to the patient's regularly scheduled appointment based on the appointment schedule for that day. All patients who stated they were responsible for instilling their own drops and had at least six months experience in administering eye drops were approached for enrollment. All subjects were consented prior to enrollment and the protocol followed the tenets of the Declaration of Helsinki; the study was approved by the Southwest Independent Institutional Review Board, Fort Worth, TX, and the Duke University Institutional Review Board. It also adhered to requirements of the Health and Insurance Portability and Accountability Act.

### 2.1. Procedures

 During the initial visit, subjects completed a demographic questionnaire that included questions about problems a patient may have with taking their glaucoma medication(s). Subjects were issued an activated Medication Event Monitoring System (MEMS) cap device and medication vial for each different hypotensive eye drop used. All subjects were educated on the purpose and correct use of the MEMS cap device and were instructed not to alter their usual, prescribed glaucoma treatment regimen. Approximately one month after enrollment, all MEMS cap devices were checked for battery life and proper recording functions. Subjects returned all MEMS caps approximately three months after enrollment.

### 2.2. Study Population

 We included all consecutive subjects with a diagnosis of glaucoma, who were using one or more topical hypotensive medications in one or both eyes. Subjects were only excluded if they did not instill their own medications, if they could not come to all follow-up study visits, or if they had a known hypersensitivity to over-the-counter artificial tears. Subjects were required to have had a complete ophthalmic exam within the preceding 6 months.

### 2.3. Measurement

#### 2.3.1. Subject Characteristics

 Self-reported race was originally measured as a categorical variable and then was dichotomized for the multivariable analyses (i.e., White, non-White). The number of glaucoma medications a subject was taking was recorded and then recoded into one versus two or more for the multivariable analyses. Length of time with the diagnosis of glaucoma was measured with the following response categories: two years or less, more than two years to less than five years, and five years or more. 

#### 2.3.2. Patient-Reported Problems with Using Glaucoma Medications

 Patients were asked how much problem or concern (none, a little, or a lot) they had in the twelve following areas: (a) side effects, (b) hard to remember all the doses, (c) hard to pay for the medications, (d) hard to get the plastic seal off a new bottle, (e) hard to open the container, (f) hard to get my refills on time, (g) hard to read the print on the container, (h) dosage times are inconvenient, (i) hard to get the drops in my eye, (j) too many drops come out at the same time, (k) drops fall on cheeks, and (l) hard to squeeze the bottle [3]. All patients except for one when they selected that they had a problem or concern stated that they had “a little” problem or concern, so each of the twelve problem areas was also recoded into dichotomous variables (no problem versus a little or a lot of a problem). The twelve problem areas were also summed to create a continuous nonweighted variable “number of problems or concerns in taking glaucoma medications.”

#### 2.3.3. Medication Adherence

 Medication adherence over a three-month period was evaluated via electronic data from the MEMS caps system (AARDEX) [16]. Adherence using MEMS caps was measured using the following formula: adherence = (number of doses used during the past three months divided by the number of prescribed doses) × 100. If the subject was on more than one glaucoma medication, an overall percent adherence variable was created by adding together the subject's adherence for each glaucoma medication and dividing it by the number of glaucoma medications the subject was using. We dichotomized the variable because the variable was skewed towards patients being highly adherent. Subjects were considered adherent if they used 80% or more of the prescribed doses, and they were classified as nonadherent if they used less than 80% of the doses prescribed. Although arbitrary, a cut-off of 80% is commonly used in adherence studies [17].

#### 2.3.4. Glaucoma Medication Adherence Self-Efficacy Questionnaire

 A 10-item general glaucoma medication adherence self-efficacy questionnaire was completed by the subjects [18]. There were three possible response choices for the self-efficacy items: not at all confident (coded as 1), somewhat confident (coded as 2), and very confident (coded as 3). Scores could range from 10 (lower self-efficacy) to 30 (higher self-efficacy). The scale has strong psychometric properties [18] and is strongly associated with adherence assessed via MEMS caps and patient self-report [18, 19]. 

#### 2.3.5. Analysis

 The a priori level of statistical significance was set at *P* < 0.05. We examined the bivariate relationships between the independent variables and between the independent and dependent variables using Pearson correlation coefficients, chi-square statistics, and *t*-tests. 

 We conducted multivariable logistic regressions to examine how patient age, years of education, gender, race, number of glaucoma medications, and length of time with glaucoma were associated with each of the twelve problems/concerns with glaucoma medications as well as with medication adherence of less than or greater than 80%. 

 Finally, we conducted a multivariable linear regression to examine how the demographic characteristics (gender, age, race, years of education, number of glaucoma medications, length of time with glaucoma) were associated with glaucoma medication adherence self-efficacy. Interaction terms between the independent variables were examined and entered into all regression models, but none were statistically significant, so they were left out of the final models.

## 3. Results

 One hundred and sixty-two subjects participated, and their demographic characteristics are presented in Table 1. Of the 56 non-White subjects, 47 identified as African American. White patients were significantly older than non-White patients (*t*-test = 2.67, *P* = 0.009). White patients had an average age of 69.4 years (standard deviation = 12.58) and non-White patients had an average age of 64.18 (standard deviation = 11.44). Non-White patients were significantly more likely to be on 2 or more medications than White patients (Pearson chi-square = 4.36, *P* = 0.037). Older patients were significantly more likely to be on 2 or more medications than younger patients (*t*-test = 2.35, *P* = 0.02). Patient race was not significantly associated with years of education.

 Patient scores on the glaucoma medication adherence self-efficacy scale ranged from 11 to 30 (mean = 27.5, standard deviation = 3.5). Patients reported an average of 2.27 problems (standard deviation = 2.19, range zero to 10). Seventy-one percent of patients expressed one or more problems in using their glaucoma medications.  Table 2 presents patient-reported problems/concerns in taking glaucoma medications.

Table 3 presents the logistic regressions examining what factors were significantly associated with some key problems/concerns that patients had in taking their glaucoma medications. Patients who were taking two or more glaucoma medications were significantly more likely to have trouble remembering to take the medications (odds ratio = 11.11, 95% confidence interval = 2.37, 52.1), side effects (odds ratio = 3.44, 95% confidence interval = 1.27, 9.29), and they felt as though the dosing times were inconvenient (odds ratio = 7.47, 95% confidence interval = 1.54, 36.07). 

 Interestingly, more educated patients were significantly more likely to report that it is hard to remember to take the medication (odds ratio = 1.23, 95% confidence interval = 1.05, 1.44), more side effects (odds ratio = 1.16, 95% confidence interval = 1.01, 1.32), and reported that dosing times are inconvenient (odds ratio = 1.19, 95% confidence interval 1.01, 1.41). Female patients reported side effects more often than male patients (odds ratio = 0.41, 95% confidence interval = 0.15, 0.96). Male patients were significantly more likely to report too many drops coming out than female patients (odds ratio = 2.20, 95% confidence interval = 1.03, 4.42).

 Eleven percent of patients were less than 80% adherent according to the MEMS devices.  Table 4 reports the logistic regression results predicting whether patients reported being 80% or more adherent to their glaucoma medications. White patients were significantly more likely to be 80% or more adherent than non-White patients (odds ratio = 3.53, 95% confidence interval 1.16, 10.76). Twenty-one percent of the non-White patients were less than 80% adherent compared to 7% of the White patients. 

Table 5 presents the linear regression results predicting glaucoma medication adherence self-efficacy. Patients who had glaucoma longer were significantly more likely to have higher glaucoma medication self-efficacy than patients who had glaucoma for a less amount of time (standardized beta = 0.22, *P* < 0.05). 

 Whether patients were 80% adherent or more was highly correlated with patient scores on the glaucoma medication adherence self-efficacy scale (*t*-test = 4.97, *P* < 0.001). Patients with higher glaucoma medication self-efficacy scores were significantly more likely to be 80% adherent or more.

## 4. Discussion

 Seventy-one percent of patients expressed one or more problems in using their glaucoma medications. This is higher than a study conducted in 2004 which found that 60% of glaucoma patients reported one or more problems [3]. We found that patients on two or more glaucoma medications were more likely to report multiple problems taking their eye drops than patients on one glaucoma medication. Patients on two or more glaucoma medications were more likely to report having difficulty with (a) remembering to take their drops, (b) side effects, (c) too many drops coming out at once, and (d) inconvenient dosing times. These findings suggest that, if possible, providers should attempt to simplify patients' regimens to fewer glaucoma medications. Research examining other disease states has shown that simplifying patients' medication regimens can improve medication adherence [20, 21].

 Eleven percent of patients took less than 80% of their prescribed glaucoma medication according to the MEMS devices. Patient race was the strongest predictor of adherence. White patients were 3 times more likely to be 80% adherent or more than non-White patients. The majority of non-White patients in our sample were African American. Our results are consistent with prior work using patient self-report to assess adherence in a Veterans' Administration population [9]. Another work using electronic monitoring of glaucoma patients treated with one specific medication also revealed non-White race as a risk for poor adherence [15]. The reasons for disparate levels of glaucoma medication adherence between White and non-White patients remain unclear. 

 We found that patients who reported more patient-reported problems had significantly lower glaucoma medication self-efficacy scores. This finding means that patients with more reported problems had less confidence in using their glaucoma medications. Providers might attempt to work to build the self-confidence of patients who report problems in using their glaucoma medications and experience low self-efficacy in using them. Providers could ask open-ended questions such as “What problems are you having in using your glaucoma medications?” or “How confident are you in using your eye drops?” to open a conversation on the topic.

Both the patient reported problems measure and the glaucoma medication adherence self-efficacy scale can be used by eye care providers to detect subjects who do not feel confident taking their glaucoma medications under certain circumstances. Providers could pinpoint the problem based on patients' responses and help address the specific challenge. Also, since the glaucoma medication adherence self-efficacy scale was significantly associated with medication adherence, the scale may help providers detect subjects who are more likely to be nonadherent with their medication regimen. The lower a patient's score on the glaucoma medication adherence self-efficacy scale, the more likely the patient is non-adherent. It is interesting that the medication adherence self-efficacy measure correlated well with MEMS device adherence. This might be because we measured self-efficacy or self-confidence in being adherent to glaucoma medications rather than asking patients how many doses they missed. Perhaps people are more likely to respond more honestly when they are asked how confident they feel when it comes to adherence rather than asking them how many doses they missed.

 We also found that patients who had glaucoma longer had significantly higher glaucoma medication adherence self-efficacy scores, which is consistent with prior research [10]. This finding illustrates the need for providers to educate patients who have had glaucoma for less time so that their self-efficacy in using the medications can be improved which could lead to improved adherence.

 We report levels of glaucoma medication adherence that are both higher [15] and lower [16] than previous reports. These differences may be in part related to the study populations, as both an academic and a private practice was included in this study as opposed to solely academic [15] or solely private practice [16].

 This study has several limitations. First, although most subjects were willing to participate, we did not track the characteristics of or number of subjects who declined to participate in the study because we could not interfere with clinic flow. Second, patients may have been more adherent during the study than usual because they knew that they were being monitored. Third, we did not assess patient motivation to adhere. Fourth, we did not record if the included subjects had had eye surgery in the past. Despite these limitations, the study provides new information about the relationship between patient race, reported problems, medication adherence, and glaucoma medication adherence self-efficacy. Further attention to the causes of poor medication adherence among specific populations may help reduce the racial disparities in visual outcomes still prevalent in glaucoma.

## Figures and Tables

**Table 1:** Subject characteristics (*N* = 162).

	Percent (*N*)
Gender	
Male	51.2 (83)
Race	
White	64.8 (105)
Black	29.0 (47)
Asian	2.5 (4)
Other	3.1 (5)
Duration of glaucoma	
2 years or less	16.0 (26)
More than 2 years to less than 5 years	19.1 (31)
5 years or more	64.2 (104)
Eye(s) affected by glaucoma	
Right	10.5 (17)
Left	1.2 (2)
Both	87.7 (142)
Number of ocular hypotensive medications used	
One	48.1 (78)
Two	38.9 (63)
Three	9.3 (15)
Four	2.5 (4)
Visual field defect severity	
Mild	61.1 (99)
Moderate	16.7 (27)
Severe	18.5 (30)

	Range; mean (standard deviation)

Age	30–92; 67.59 (12.41)
Years of education	5–30; 15.15 (3.64)

**Table 2:** Problems/difficulties in taking glaucoma medications (*N* = 162).

	Percent (*N*)
Drops fall on cheek	40.1 (65)
Problem paying	25.3 (41)
Too many drops come out	29.0 (47)
Can't get drops in eyes	18.5 (30)
Hard to read print	25.9 (42)
Side effects	17.3 (28)
Hard to squeeze the bottle	12.3 (20)
Difficult to get seal off bottle	13.6 (22)
Difficult to get refills	11.7 (19)
Difficulty in remembering	12.3 (20)
Hard to open bottle	6.2 (10)
Dosage times inconvenient	9.9 (16)

**Table 3:** Multivariable logistic regression results predicting problems with glaucoma medications (*N* = 159).

Independent variables	Hard to remember	Side effects	Too many drops come out	Dosing times are inconvenient	Difficult to read the print
	OR (95% CI)	OR (95% CI)	OR (95% CI)	OR (95% CI)	OR (95% CI)
Age	0.99 (0.94, 1.04)	0.98 (0.94, 1.02)	1.01 (0.98, 1.05)	0.98 (0.93, 1.03)	0.98 (0.95, 1.01)
Years of education	1.23 (1.05, 1.44)*	1.16 (1.01, 1.32)*	1.01 (0.92, 1.12)	1.19 (1.01, 1.41)*	0.99 (0.89, 1.10)
Gender: male	0.97 (0.34, 2.75)	0.41 (0.15, 0.96)*	2.20 (1.03, 4.42)*	0.43 (0.13, 1.42)	0.71 (0.34, 1.49)
Race: White	0.90 (0.29, 2.76)	0.52 (0.21, 1.30)	1.03 (0.47, 2.23)	1.10 (0.32, 3.85)	0.59 (0.27, 1.30)
No. of glaucoma medications	11.11 (2.37, 52.1)**	3.44 (1.27, 9.29)*	2.20 (1.04, 4.66)*	7.47 (1.54, 36.07)*	0.94 (0.43, 2.04)
How long have had glaucoma	1.13 (0.79, 1.62)	0.89 (0.68, 1.17)	0.92 (0.74, 1.15)	1.98 (1.02, 3.88)*	0.77 (0.62, 0.96)*

*P < 0.05

**P < 0.01

***P < 0.001

OR: odds ratio, CI: confidence interval.

**Table 4:** Logistic regression predicting patient being 80% adherent or more according to Medication Event Monitoring System (MEMS) adherence (*N* = 159).

Independent variables	Adherence
	Odds ratio (95% CI)
Age	1.02 (0.97, 1.07)
Years of education	1.11 (0.94, 1.29)
Gender: female	1.82 (0.61, 5.37)
Race: White	3.53 (1.16, 10.76)*
No. of glaucoma medications	0.63 (0.20, 1.96)
How long have had glaucoma	1.22 (0.90, 1.66)
Total number of patient-reported problems	0.93 (0.74, 1.17)

*P < 0.05; CI: confidence interval.

**Table 5:** Multivariable linear regression predicting glaucoma medication adherence self-efficacy (*N* = 159).

Independent variables	Glaucoma medication adherence self-efficacy
	Standardized beta
Age	−0.03
Years of education	−0.10
Gender: female	0.15
Race: White	0.01
No. of glaucoma medications	0.01
How long have had glaucoma	0.22*
Total number of patient-reported problems	−0.42***

*P < 0.05; **P < 0.01; ***P < 0.001.

## References

[1] Quigley HA, Broman AT (2006). The number of people with glaucoma worldwide in 2010 and 2020. *British Journal of Ophthalmology*.

[2] Lichter PR, Musch DC, Gillespie BW (2001). Interim clinical outcomes in the collaborative initial glaucoma treatment study comparing initial treatment randomized to medications or surgery. *Ophthalmology*.

[3] Sleath B, Robin AL, Covert D, Byrd JE, Tudor G, Svarstad B (2006). Patient-reported behavior and problems in using glaucoma medications. *Ophthalmology*.

[4] Nordstrom BL, Friedman DS, Mozaffari E, Quigley HA, Walker AM (2005). Persistence and adherence with topical glaucoma therapy. *American Journal of Ophthalmology*.

[5] Schwartz GF, Platt R, Reardon G, Mychaskiw MA (2007). Accounting for restart rates in evaluating persistence with ocular hypotensives. *Ophthalmology*.

[6] Friedman DS, Okeke CO, Jampel HD (2009). Risk factors for poor adherence to eyedrops in electronically monitored patients with glaucoma. *Ophthalmology*.

[7] Patel SC, Spaeth GL (1995). Compliance in patients prescribed eyedrops for glaucoma. *Ophthalmic Surgery*.

[8] Muir KW, Santiago-Turla C, Stinnett SS (2006). Health literacy and adherence to glaucoma therapy. *American Journal of Ophthalmology*.

[9] Sleath B, Ballinger R, Covert D, Robin AL, Byrd JE, Tudor G (2009). Self-reported prevalence and factors associated with nonadherence with glaucoma medications in veteran outpatients. *American Journal Geriatric Pharmacotherapy*.

[10] Sleath B, Blalock SJ, Stone JL (2012). Validation of a short version of the glaucoma medication self-efficacy questionnaire. *British Journal of Ophthalmology*.

[11] Melfi CA, Croghan TW, Hanna MP, Robinson RL (2000). Racial variation in antidepressant treatment in a Medicaid population. *Journal of Clinical Psychiatry*.

[12] Krishnan JA, Diette GB, Skinner EA, Clark BD, Steinwachs D, Wu AW (2001). Race and sex differences in consistency of care with National Asthma Guidelines in managed care organizations. *Archives of Internal Medicine*.

[13] Monane M, Bohn RL, Gurwitz JH, Glynn RJ, Levin R, Avorn J (1996). Compliance with antihypertensive therapy among elderly medicaid enrollees: the roles of age, gender, and race. *American Journal of Public Health*.

[14] Congdon N (2004). Causes and prevalence of visual impairment among adults in the United States. *Archives of Ophthalmology*.

[15] Okeke CO, Quigley HA, Jampel HD (2009). Adherence with topical glaucoma medication monitored electronically. The travatan dosing aid study. *Ophthalmology*.

[16] Robin AL, Novack GD, Covert DW, Crockett RS, Marcic TS (2007). Adherence in glaucoma: objective measurements of once-daily and adjunctive medication use. *American Journal of Ophthalmology*.

[17] Osterberg L, Blaschke T (2005). Adherence to medication. *New England Journal of Medicine*.

[18] Sleath B, Blalock S, Covert D (2011). The relationship between glaucoma medication adherence, eye drop technique, and visual field defect severity. *Ophthalmology*.

[19] Sleath B, Blalock SJ, Robin A (2010). Development of an instrument to measure glaucoma medication self-efficacy and outcome expectations. *Eye*.

[20] Roter DL, Hall JA, Merisca R, Nordstrom B, Cretin D, Svarstad B (1998). Effectiveness of interventions to improve patient cmpliance a meta-analysis. *Medical Care*.

[21] Haynes RB, McDonald HP, Garg AX (2002). Helping patients follow prescribed treatment: clinical applications. *Journal of the American Medical Association*.

